# Liver Transplantation in Cases with Acute Liver Failure

**DOI:** 10.5005/jp-journals-10018-1139

**Published:** 2016-07-09

**Authors:** Mustafa Yalçin, Göksel Bengİ, Mesut Akarsu, Tarkan İnek, İbrahim Astarcioşlu

**Affiliations:** 1 Department of Gastroenterology, Dokuz Eylul University Faculty of Medicine, İzmir, Turkey; 2 Department of General Surgery, Dokuz Eylul University Faculty of Medicine, İzmir, Turkey

**Keywords:** Acute liver failure, Liver transplantation, Treatment of ALF

## Abstract

**Background:**

Acute liver failure (ALF) is a rare, life-threatening clinical condition that is characterized by severe hepatocellular necrosis, jaundice, coagulopathy and encephalopathy. The aim of this study was to evaluate patients who underwent liver transplantation at Dokuz Eylul University of Medicine Faculty (DEUMF) due to ALF.

**Materials and methods:**

The patients who underwent liver transplantation at DEUMF due to ALF were evaluated retrospectively.

**Results:**

All of the liver transplantations performed in this study were successful; toxicity was the major cause of ALF in these patients (84%).

**Conclusion:**

Results of this study may not be generalizable to all of Turkey since the patients included in this study were only from one region. However, our study is in accordance with others that show that liver transplantation is a very safe and effective method for the treatment of ALF.

**How to cite this article:**

Yalçin M, Bengİ G, Akarsu M, İnek T, Astarcioşlu İ. Liver Transplantation in Cases with Acute Liver Failure. Euroasian J Hepato-Gastroenterol 2015;5(2):80-82.

## INTRODUCTION

Acute liver failure (ALF) is a rare, life-threatening clinical condition that is characterized by severe and instant hepatocellular necrosis, jaundice, coagulopathy and encephalopathy. Acute liver failure cases have no history of liver disease and the symptoms of ALF appear within 26 weeks.^1,2^ In developed counties, the major cause of ALF is toxicity; in undeveloped countries, ALF is caused by viral factors.

Acute liver failure is related to high morbidity and mortality. Interactions between the patient, genetics, the cause of the hepatic injury and the liver play a role in the prognosis of ALF.^3^ Currently, the survival of ALF patients without liver transplantation is unclear. The MELD score, INR and King’s College criteria are methods of predicting survival. Recently, there has been limited development in the prognostic scoring system and laboratory tests. The most practical scoring system is the King’s College criteria, which predicts mortality caused by paracetamol and other causes.

A meta-analysis consisting of 18 studies and 1105 patients indicated that the sensitivity, specificity and odds ratio of the King’s College criteria in ALF caused by nonparacetamol causes were 68 [95% confidence interval (CI) 59-77], 82% (95% CI 75-88) and 12.6 (95% CI 6.5-26.1), respectively.^4^ Today, 45% of adult ALF patients recover spontaneously, 25% undergo liver transplantation, and 30% of the adult patients pass before the liver transplantation due to absolute contraindications. In children with ALF, 56% recover spontaneously, 30% undergo liver transplantation, and 13% pass before the liver transplantation.^5^

The aim of this study is to evaluate the patients who underwent liver transplantation at Dokuz Eylul University of Medicine Faculty (DEUMF) due to ALF.

## MATERIALS AND METHODS

The patients who underwent liver transplantation at DEUMF due to ALF were evaluated retrospectively. Sex, age, preoperative biochemical test results, causes of ALF and the conditions of the donors (alive or cadaver) were recorded. Data were examined using Microsoft Excel software.

## RESULTS

Five of the cases (38.4%) who underwent liver transplantation at DEUMF due to ALF were male and eight (61.6%) were female. The mean age of the subjects was 29 (5-61) years. Three of the donors (23%) were cadaver and 10 of them (77%) were living. The mean values of the patients were as follows: Total Bilirubin: 31 mg/dl, AST: 766U/L, ALT: 787U/L, INR: 3.2, albumin: 3 gr/dl, MELD score: 31.5, encephalopathy stage: 2.5. When the etiology of ALF was examined, it was found that ALF was caused by drugs in six patients, caused by fireworks (due to the content of phosphorus) in two patients, caused by mushrooms in two patients, and caused by herbalin (herba centaurii) in one patient. One of the patients underwent liver transplantation due to autoimmune hepatitis and another for cryptogenic ALF (Tables 1 and 2). When we examine drug induced hepatotoxicity cases, Ornidazole and amoxicillin-clavulanate were found to be responsible for one patient. On the other hand, active substance could not be identified due to multiple drug use. Liver transplantation was successful in the treatment of all patients.

**Table 1:** **Table 1:** Characteristics of the cases

*Cases (N)*		*Sex*		*Age (years)*		*Factor*		*Donor*	
N1		F		5		Autoimmune		Alive	
N2		F		22		Cryptogenic		Alive	
N3		F		47		Toxic		Cadaver	
N4		M		20		Toxic		Alive	
N5		M		28		Toxic		Alive	
N6		F		5		Toxic		Alive	
N7		F		8		Toxic		Alive	
N8		M		33		Toxic		Alive	
N9		M		43		Toxic		Alive	
N10		F		36		Toxic		Alive	
N11		F		61		Toxic		Cadaver	
N12		F		44		Toxic		Cadaver	
N13		M		28		Toxic		Alive	
Total 13		61% F		Average age		11 Toxic		23%	
		39% M		29.2		1 Autoimmune		Cadaver	
						1 Cryptogenic		77% Alive	

F: Female; M: Male

**Table 2:** **Table 2:** Laboratory test results of cases

*Case*		*AST (u/l)*		*ALT (u/l)*		*T. Bil (mg/dl)*		*Cr. (mg/dl)*		*Na (meq/l)*		*Alb (g/dl)*		*INR*		*MELD*		*Encephalopathy grade*		*Assit*	
N1		57		73		17.29		0.52		143		5.3		2.933		29		4		None	
N2		622		756		20.56		0.5		139		2.3		3.406		32		4		Low	
N3		1507		1668		45		0.2		139		3.4		4.42		37		4		Low	
N4		625		504		48		1		137		2.8		2.9		33		1		Low	
N5		1723		1967		42		0.6		138		2.7		6.05		41		3		None	
N6		2210		1188		26.5		0.2		142		3.4		2.3		28		3		Low	
N7		1747		1150		3.2		1		132		1.8		2.3		20		3		None	
N8		126		197		43.92		0.56		137		3		2.55		31		1		Low	
N9		87		1540		19.92		0.81		144		3.2		3.18		31		3		Low	
N10		622		659		26.06		0.33		140		2.7		2.84		30		1		None	
N11		120		70		39.01		0.63		137		2.8		5		38		2		None	
N12		205		134		46.7		0.2		133		2.9		2.18		30		1		Low	
N13		313		331		33.8		0.39		149		3.1		2.5		30		3		None	
Total 13		766.4		787.4		31.6		0.5		139.2		3.0		3.2		31.5		2.5			

F: Female; M: Male; T. bil: Total bilirubin; Cr: Creatine; Alb: Albumine; MELD: Model for end-stage liver disease

## DISCUSSION

Acute liver failure is a rare, life-threatening clinical condition that is characterized by severe and instant hepatocellular necrosis, jaundice, coagulopathy and encephalopathy. Acute liver failure cases have no history of liver disease, and the symptoms of ALF appear within 26 weeks.^1,2^ Toxicity is the major cause of ALF in developed countries, while viral factors are the major cause in developing countries. Kayaalp et al analyzed 308 Turkish patients and found that the most common causes of ALF were hepatitis A in children (20.9%) and hepatitis B in adults (34.7%). Eighteen percent of ALS cases are due to cryptogenic causes, while 14% are due to metabolic causes. Wilson disease is the most common cause of metabolic diseases, while toxic liver failure is most often due to mushroom poisoning (13%). Poisoning from fireworks and phosphorus are regional factors, while anti-tuberculosis agents (3.2% of ALF causes) are the most common cause of drug-based ALF. Paracetamol is responsible for only 0.7% of ALF cases.^6^ In our current study, toxicity was the major cause of ALF in the patients who underwent liver transplantation (84%). None of the patients who underwent liver transplantation had ALF caused by viral factors. We believe that the results of this study do not reflect the patient profile of all of Turkey, but reflects only the profile of the patients who applied to our hospital.

Acute liver failure is related to high morbidity and mortality. Interactions between the patient, genetics, the cause of the hepatic injury play a role in the prognosis of ALF.^3^ Today, 45% of adult patients recover spontaneously, 25% undergo liver transplantation, and 30% are lost before the liver transplantation due to absolute contraindications. On the other hand, 56% of children with ALF recover spontaneously, 30% undergo liver transplantation, and 13% are lost before the liver transplantation.^5^ In our hospital, 13 cases of acute liver failure were treated via liver transplantation (three from cadaver, 10 from living donor). Liver transplantation is a very successful method for the treatment of ALF.

Results of this study indicate that toxicity is the major cause of ALF in patients who underwent liver transplantation at DEUMF. However, this result is not in accordance with the general situation in Turkey. Our current results only reflect the characteristics of our region. However, liver transplantation was successful in all patients, and there were no complications during or after the surgery. As previous studies have shown, liver transplantation is a very safe and effective method for the treatment of ALF.
